# Altered hippocampal expression of glutamate receptors and transporters in GRM2 and GRM3 knockout mice

**DOI:** 10.1002/syn.20553

**Published:** 2008-11

**Authors:** Louisa Lyon, James NC Kew, Corrado Corti, Paul J Harrison, Philip WJ Burnet

**Affiliations:** 1 Department of Psychiatry, University of Oxford, Warneford HospitalOxford OX3 7JX, United Kingdom; 2 Psychiatry DTG, GlaxoSmithKline, New Frontiers Science ParkThird Avenue, Harlow, Essex CM195AW, United Kingdom; 3 Psychiatry Centre of Excellence for Drug Discovery, GlaxoSmithKlineVia Fleming 4, Verona, Italy

**Keywords:** group II metabotropic glutamate receptor, mGlu2, mGlu3, hippocampus, mRNA, NMDA receptor, glutamate transporter

## Abstract

Group II metabotropic glutamate receptors (mGluR2 and mGluR3, also called mGlu2 and mGlu3, encoded by *GRM2* and *GRM3*, respectively) are therapeutic targets for several psychiatric disorders. *GRM3* may also be a schizophrenia susceptibility gene. mGluR2^−/−^ and mGluR3^−/−^ mice provide the only unequivocal means to differentiate between these receptors, yet interpretation of in vivo findings may be complicated by secondary effects on expression of other genes. To address this issue, we examined the expression of NMDA receptor subunits (NR1, NR2A, NR2B) and glutamate transporters (EAAT1-3), as well as the remaining group II mGluR, in the hippocampus of mGluR2^−/−^ and mGluR3^−/−^ mice, compared with wild-type controls. mGluR2 mRNA was increased in mGluR3^−/−^ mice, and vice versa. NR2A mRNA was increased in both knockout mice. EAAT1 (GLAST) mRNA and protein, and EAAT2 (GLT-1) protein, were reduced in mGluR3^−/−^ mice, whereas EAAT3 (EAAC1) mRNA was decreased in mGluR2^−/−^ mice. Transcripts for NR1 and NR2B were unchanged. The findings show a compensatory upregulation of the remaining group II metabotropic glutamate receptor in the knockout mice. Upregulation of NR2A expression suggests modified NMDA receptor signaling in mGluR2^−/−^ and mGluR3^−/−^ mice, and downregulation of glutamate transporter expression suggests a response to altered synaptic glutamate levels. The results show a mutual interplay between mGluR2 and mGluR3, and also provide a context in which to interpret behavioral and electrophysiological results in these mice.

## INTRODUCTION

Group II metabotropic glutamate receptors comprise mGluR2 (mGlu2, encoded by *GRM2*) and mGluR3 (mGlu3, encoded by *GRM3*). Both are G–protein coupled receptors, whose activation inhibits adenylate cyclase and decreases cAMP formation. Their primary functions are thought to be as inhibitory autoreceptors and thence modulation of glutamatergic signaling (Anwyl, 1999; Cartmell and Schoepp, 2000); they also act as heteroceptors modulating the release of other transmitters including dopamine and GABA. There is considerable psychiatric interest in these receptors, for two main reasons. First, they are therapeutic targets for several disorders (Harrison, 2008; Moghaddam, 2004; Swanson et al., 2005), with recent clinical trials showing positive results in schizophrenia (>Patil et al., 2007) and in generalized anxiety disorders (Dunayevich et al., 2007). Second, mGluR3 is a putative candidate gene for schizophrenia and is implicated in its pathophysiology (Corti et al., 2007a; Egan et al., 2004; Harrison et al., in press; Sartorius et al., 2008; Tan et al., 2007) as part of a broader glutamatergic dysfunction (Harrison and Weinberger, 2005; Konradi and Heckers, 2003).

Given these findings, it becomes important to distinguish the properties of mGluR2 from those of mGluR3, a distinction that has been hampered by the lack of adequately selective ligands and antibodies. Nevertheless, several differences are known (for review, see Harrison et al., in press), including cellular and subcellular localization (Tamaru et al., 2001), expression profile across brain development (Catania et al., 1994), the presence of splice variants (Sartorius et al., 2006), and interactions with protein phosphatase 2C (Flajolet et al., 2003). These findings are complemented recently by data in mGluR2^−/−^ and mGluR3^−/−^ mice. For example, mGluR2^−/−^ mice show reduced mossy fiber long–term depression (Yokoi et al., 1996) and locomotor hyperactivity (Morishima et al., 2005), whereas mGluR3^−/−^ mice show enhanced hippocampal c–Fos expression (Linden et al., 2006) and vulnerability to NMDA–induced neurotoxicity (Corti et al., 2007b). However, conclusions drawn from knockout mice may be complicated by secondary changes in expression of other genes, which may compensate for, enhance, or otherwise modify, the effect of the gene deletion. Currently, the molecular phenotypes of mGluR2^−/−^ and mGluR3^−/−^ mice remain unknown. This study has therefore examined the expression of key genes involved in glutamatergic neurotransmission in both knockout mice compared with their respective wild–type littermates. We focused on the hippocampus, as a structure critically involved in schizophrenia (Harrison, 2004) and cognition (Martin and Clark, 2007; Sweatt, 2004), and in which mGluR function has previously been examined in detail. The targeted genes included the remaining group II mGluR, to assess the possibility of compensatory upregulation, as well as NMDA receptors (NR1, NR2A, NR2B subunits) and glutamate transporters (EAAT1 [GLAST], EAAT2 [GLT–1], and EAAT3 [EAAC1]). These receptors and transporters were measured because there is already pharmacological evidence linking them with mGluR2 and/or mGluR3 (e.g., Aronica et al., 2003; Cartmell et al., 1999; Gegelashvili et al., 2000; Krystal et al., 2005; Moghaddam and Adams, 1998), and we predicted a change in their expression as a correlate of the altered glutamate signaling characteristics that likely accompany the genetic deletion of a group II mGluR. In each case, quantification of the encoding mRNA by in situ hybridization was used; in the case of EAAT1 and EAAT2 we complemented these results with immunoautoradiographic assessment of the protein. Our results show that deletion of mGluR2 or mGluR3 produces similar but not identical effects on expression of these glutamatergic genes, and illustrate the importance of both group II mGluRs in hippocampal glutamate signaling and thence, presumably, in hippocampal functioning. Some of the data have been presented in abstracts (Lyon et al., 2006, 2007).

## MATERIALS AND METHODS

### Animals and tissue sectioning

mGluR2^−/−^ and mGluR3^−/−^ mice were generated on a C57Bl/6 × 129SVj background, as described (Corti et al., 2004, 2007a; Yokoi et al., 1996). Brains from six male 12–week–old mGluR2^−/−^ mice and six wild–type littermates, plus six male 12–week–old mGluR3^−/−^ mice and six wild–type littermates, were snap frozen by immersion in chilled isopentane. Brains were stored at −80°C. Unfixed coronal sections (12 μm) containing the dorsal hippocampus were collected on a Shandon cryostat at −20°C. Sections were collected on Superfrost Plus slides (VWR, Lutterworth, UK), three per slide, and stored at −70°C.

### In situ hybridization histochemistry

In situ hybridization histochemistry was carried out as described by Eastwood et al. (2007). Pretreatment consisted of: freshly prepared 4% paraformaldehyde in phosphate–buffered saline (PBS) (5 min); (PBS rinse); 0.1 M triethanolamine–NaCl solution containing 0.25% acetic anhydride added immediately prior to use (10 min); 70% ethanol (1 min); 80% ethanol (1 min); 95% ethanol (2 min); 100% ethanol (1 min); chloroform (10 min); 100% ethanol (1 min); 95% ethanol (1 min). Sections were air7–dried and stored at −20°C. Oligonucleotide probes were synthesized (Sigma, Dorset, UK) (Table I) and 3′ end–labeled with [^35^S]dATP using the enzyme terminal deoxynucleotidyl transferase (TdT) (Promega, Southampton, UK). Labeled probe was purified from unincorporated nucleotides using Nick Columns (Amersham, Bucks, UK) according to manufacturer's instructions. Sections were incubated overnight at 35°C in 225 μl hybridization buffer [4× sodium saline citrate (SSC), 50% deionized formamide, 10% dextran sulfate, 25 mM sodium phosphate (pH 7.0), 5× Denhardt's solution, 200 μg/ml salmon sperm DNA, 100 μg/ml poly(A), 1 mM sodium pyrophosphate, 120 μg/ml heparin] containing 3× 10^6^ counts per minute of labeled probe and 20 μM dithiothreitol. Posthybridization washes were conducted in 1× SSC for 3× 20 min at 55°C, followed by 2× 60 min at room temperature. Hybridized sections were apposed to Kodak Biomax MS film together with ^14^C microscales (GE Healthcare, Little Chalfont, UK) for an optimal length of time determined by trial experiments (Table I).

**TABLE 1:** Nucleotide sequences of probes used for in situ hybridization histochemistry, and exposure times against autoradiographic film

Gene	GenBank accesssion number	Oligodeoxynucleotide sequence (5′ to 3′)	Exposure time(days)
mGluR2	XM_001475814.1	GGT GAA GAG CAC AGC CAC ACG GGC GCT GGG CTT CT	12
mGluR3	NM_181850.2	TTG CCA CCT GTA TGG AAA CAC TGC TGT ACG AAC CC	21
EAAT1	NM_148938.2	GTA GTA GTC ATG TAA TAG ACT ACA GCG CGC ATC CCC	12
EAAT2	NM_011393.2	GTA CCT TGC ACT CAT CTA TTA CGA CAG AGT TGT GT	5
EAAT3	NM_009199.2	TAG TGA GTT CCA GGA TAT CCA GGG CTA TGT AGA GA	4
NR1	NM_008169	GTA GAC CTG GCT GGA GAT GAG GTC CTC GCA CAC CGA CAG AGC CAT	4
NR2A	NM_008170	AGA AGG CCC GTG GGA GCT TTC CCT TTG GCT AAG TTTC	4
NR2B	NM_008171.3	GGG CCT CCT GGC TCT CTG CCA TCG GCT AGG CAC CTG TTG TAA CCC	8

### Immunoautoradiography

Sections were fixed in 4% paraformaldehyde/PBS for 5 min, and then washed twice in PBS. Nonspecific binding was blocked by incubating the slides in 200 μl of normal donkey serum (Sigma, Dorset, UK), diluted 1:10 in PBS with 0.3% Triton X–100 (PBS–T), for 30 min at room temperature. Sections were incubated overnight at 4°C with rabbit polyclonal anti–EAAT1 (Abcam, Cambridge, UK) diluted 1:200 in PBS–T, or with rabbit polyclonal anti-EAAT2 (Abcam) diluted 1:200 with PBS–T. Omission of primary antibody was used as a control. After three PBS washes to remove unbound primary, sections were incubated with 0.2 μCi/ml [^35^S]–labeled donkey antirabbit IgG (Amersham) at 1:500 in PBS–T and 1% donkey serum, for 1 h at room temperature. Following washes in PBS and MilliQ Ultrapure water to remove unbound secondary, slides were left to air–dry, before being placed against Kodak Biomax MS film with ^14^C microscales for 7 days.

### Image and statistical analysis

Autoradiographic films were developed under safelight conditions and measured using the MCID Elite v7.0 image analysis system (Interfocus, Haverhill, UK). Optical density values were calibrated to ^35^SnCi/g tissue equivalents using the ^14^C microscales. In the case of the neuronally expressed mRNAs, namely EAAT3 and the NMDAR subunits, measurements were taken over the stratum granulosum of the dentate gyrus, and over the stratum pyramidale of CA3 and CA1 subfields. In contrast, EAAT1 and EAAT2 mRNA and immunoreactivity were measured over a broadband distributed across the various strata of each subfield, reflecting the diffuse localization of these glial-expressed genes.

All data were explored for normality using the Kolmogorov–Smirnov test (in SPSS for Windows, v15.0). Group differences (knockout vs. wild-type) in the hippocampal expression of each gene were analyzed using ANOVA across all subfields examined; if a significant effect of genotype or genotype–by–subfield interactions were observed, separate analyses were performed in each subfield. Experiments on the mGluR2^−/−^ and mGluR3^−/−^ mice were conducted at different times (and compared with separate control groups), and were therefore analyzed separately.

## RESULTS

The regional hippocampal distribution of each transcript was as reported previously: mGluR2 (Ohishi et al., 1993a), mGluR3 (Ohishi et al., 1993b), EAAT1 and EAAT2 (Ingram et al., 2001), EAAT3 (Kanai and Hediger, 1992), and NMDAR subunits (Monyer et al., 1992). Hybridization in the presence of excess unlabeled probe produced minimal background signal (not shown). The distribution of EAAT1 (Schmitt et al., 1997) and EAAT2 (Rothstein et al., 1994) immunoreactivity was also in agreement with previous findings and western blotting confirmed that the antibodies we used produced a single band of the expected ∼65 kDa (EAAT1) and ∼73 kDa (EAAT2) in mouse brain (data not shown).

### mGluR2 and mGluR3 mRNA

**Fig. 1:**
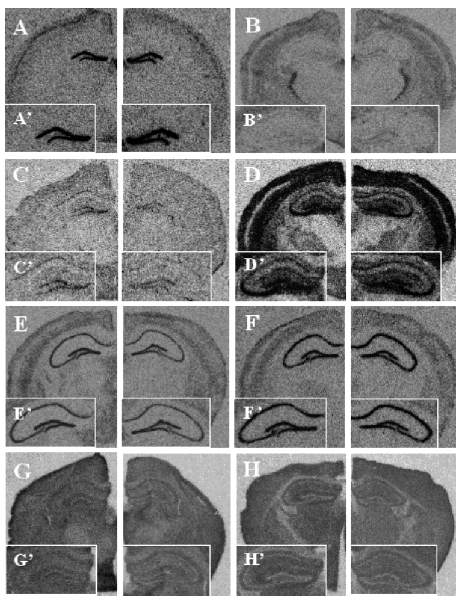
**A:** mGluR2 mRNA distribution in wild types (left-hand panel); mGluR2 mRNA signal is increased in mGluR3^−/−^ mice (right-hand panel). **B:** mGluR3 mRNA in wild types (left); mGluR3 mRNA is increased in mGluR2^−/−^ mice (right). **C:** EAAT1 (GLAST) mRNA in wild types (left); EAAT1 (GLAST) mRNA is decreased in mGluR3^−/−^ mice (right). **D:** EAAT2 (GLT-1) mRNA in wild types (left); EAAT2 (GLT-1) mRNA is unchanged in mGluR3^−/−^ mice (right) and in mGluR2^−/−^ mice (not shown). **E:** EAAT3 (EAAC1) mRNA in wild types (left); EAAT3 (EAAC1) mRNA is decreased in mGluR2^−/−^ mice (right). **F:** NR2A mRNA in wild types (left); NR2A mRNA is increased in mGluR2^−/−^ mice (right) and in mGluR3^−/−^ mice (not shown). **G:** EAAT1 (GLAST) protein in wild types (left); EAAT1 (GLAST) protein is decreased in mGluR3^−/−^ mice (right). **H:** EAAT2 (GLT-1) protein in wild types (left); EAAT2 (GLT-1) protein is decreased in mGluR3^−/−^ mice (right) and in mGluR2^−/−^ mice (not shown). Inset panels display hippocampal enlargements.

Hybridization signals for mGluR2 (Fig. 1A) and mGluR3 (Fig. 1B) mRNA were present mainly over the dentate gyrus, and were only quantified over this subfield.

#### mGluR2 mRNA

**Fig. 2:**
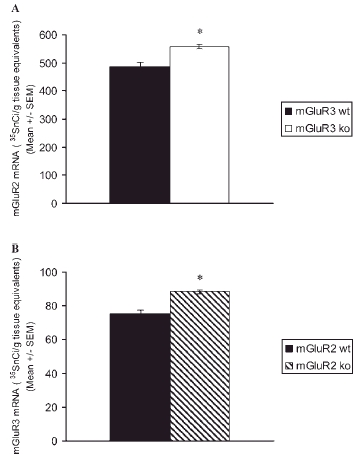
mGluR2 mRNA **(A)** is increased in the dentate gyrus of mGluR3−/− mice (n=6) compared to wild types (n=6), whereas mGluR3 mRNA **(B)** is increased in the dentate gyrus of mGluR2−/− mice (n=4) compared to wild types (n=4).

mGluR2 mRNA was increased in the mGluR3^−/−^ mice compared with their wild–type littermates (*F*_(1,10)_ = 14.81; *P* = 0.003; Figs. 1A and 2A).

#### mGluR3 mRNA

mGluR3 mRNA was increased in the mGluR2^−/−^ mice compared with their wild–type littermates (*F*_(1,6)_ = 23.14; *P* = 0.003) (Figs. 1B and 2B).

### Glutamate transporter mRNAs

#### EAAT1 (GLAST) mRNA

For EAAT1 mRNA, in mGluR2^−/−^ mice there was no effect of genotype and no genotype–by–subfield interaction (both *F* < 1, *P* > 0.20). By contrast, mGluR3^−/−^ mice showed a main effect of genotype (*F*_(1,20)_ = 15.22; *P* < 0.001) as well as a trend genotype–by–subfield interaction (*F*_(1,20)_ = 3.68; *P* = 0.070). Subsequent analysis revealed that EAAT1 mRNA was reduced in both the dentate gyrus (*F*_(1,10)_ = 9.77; *P* = 0.011) and CA1 (*F*_(1,10)_ = 7.38; *P* = 0.022) of mGluR3^−/−^ mice compared with their wild-type littermates (Table II; Fig. 1C).

**TABLE II:** Hippocampal expression of *EAAT1 (GLAST) mRNA* and protein, *EAAT2 (GLT–1)* mRNA and protein, *EAAT3 (EAAC1)* mRNA,and NR1, NR2A, and NR2B mRNA in mGluR2−/− and mGluR3−/− mice

			mGluR2		mGluR3	
				
Gene			wt(n=5)	ko(n=4–6)	wt(n=6)	ko(n=6)
EAATI	mRNA^a^	DG	279±13	264±17	259±14	210±*
		CAI	159±5	159±11	142±5	125±4*
	protein^b^	DG	1210±123	1206±75	453±22	375±18*
EAAT2	mRNA	CAI	1343±92	1283±85	460±	372±18*
		DG	1357±99	1339±51	1425±88	1479±100
		CA3	2455±29	2588±	2141±151	2147±187
		CAI	956±44	944±32	745±40	722±49
	protein^c^	DG	1160±67	1259±42	1733±58	1542±53*
		CA3	1037±62	1070±24	1591±42	1454±38*
		CAI	1081±33	1087±54	1699±51	1459±43**
EAAT3	mRNA^d^	DG	3148±351	2046±362	2579±276	2684±229
		CA3	1612±382	941±122	1652±286	1746±252
		CAI	1741±144	1058±104**	1917±277	1990±253
NRI	mRNA^e^	DG	1449±45	1539±49	1485±68	1534±48
		CA3	1454±78	1289±47	1337±77	1456±33
		CAI	1377±58	1554±92	1535±65	1593±37
NR2A	mRNA^f^, ^g^	DG	811±17	1011±49*	1326±34	1507±53*
		CA3	723±55	693±49	1061±34	1172±33*
		CAI	979±65	1104±71	1467±69	1611±80
NR2B	mRNA	DG	913±26	948±31	1087±45	1037±20
		CA3	840±40	784±54	912±30	886±37
		CAI	1111±42	1184±20	1344±80	1378±54

Values are mean ^35^SnCi/g tissue equivalents ±SEM

wt, wilg type;ko, knockout

a Main effect of genotype in mGluR3^−/−^ mice, *P*<0.001(univariate ANOVA)

b Main effect of genotype in mGluR3^−/−^ mice, *P*=0.001(univariate ANOVA)

c Main effect of genotype in mGluR3^−/−^ mice, *P*<0.001(univariate ANOVA)

d Main effect of genotype in mGluR3^−/−^ mice, *P*<0.001(univariate ANOVA)

e Genotype by subfield interaction in mGluR2^−/−^mice, *P*=0.033(univariate ANOVA)

f Main effect of genotype in mGluR2^−/−^ mice, *P*=0.045 (univariate ANOVA)

g Main effect of genotype in mGluR3^−/−^ mice, *P*=0.002(univariate ANOVA)

* *P*<0.05

** *P*<0.01, post–hoc comparisons (one–way ANOVA)

#### EAAT2 (GLT-1) mRNA

For EAAT2 mRNA there was no effect of genotype or no genotype–by–subfield interaction, in either mGluR2^−/−^ or mGluR3^−/−^ mice (all *F* < 1, P > 0.20) (Table II; Fig. 1D).

#### EAAT3 (EAAC1) mRNA

**Fig. 3:**
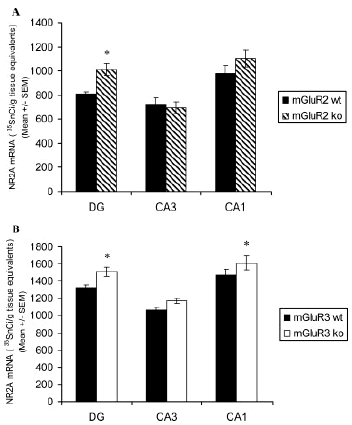
NR2A mRNA is increased in the hippocampus of both (**A**) mGluR2^−/−^ (n = 6) compared to wild type (n = 6) and (**B**) mGluR3^−/−^ (n = 5) compared to wild type (n = 5) mice.

In mGluR2^−/−^ mice, EAAT3 mRNA showed a main effect of genotype (*F*_(1,21)_ = 14.80; *P* < 0.001) but no interaction with subfield (*F* < 1; *P* > 0.20). One–way ANOVA confirmed that EAAT3 mRNA was reduced throughout the mGluR2^−/−^ hippocampus, significantly in CA1 (*F*_(1,7)_ = 13.27; *P* = 0.008) and at trend significance in dentate gyrus (*F*_(1,7)_ = 4.69; *P* = 0.067) and CA3 (*F*_(1,7)_ = 3.98; *P* = 0.086) (Fig. 3E). EAAT3 mRNA was unaltered in mGluR3^−/−^ mice, with no effect of genotype and no genotype–by–subfield interaction (both *F* < 1; *P* > 0.20) (Table II;).

### Glutamate transporter proteins

#### EAAT1 (GLAST)

Results for EAAT1 immunoreactivity were similar to those for EAAT1 mRNA. Thus, in mGluR2^−/−^ mice, there was no effect of genotype and no genotype–by–subfield interaction (both *F* < 1; *P* > 0.20). In mGluR3^−/−^ animals, there was an effect of genotype (*F*_(1,20)_ = 15.62; *P* = 0.001), but no genotype-by-subfield interaction (*F* < 1; *P* > 0.20). EAAT1 immunoreactivity was reduced in a fairly uniform manner throughout the mGluR3^−/−^ hippocampus (dentate gyrus: *F*_(1,10)_ = 7.60; *P* = 0.02; CA1: *F*_(1,10)_ = 8.03; *P* = 0.018) (Table II; Fig. 1G).

#### EAAT2 (GLT–1)

EAAT2 immunoreactivity was not changed in mGluR2^−/−^ animals, with no effect of genotype, and no genotype-by-subfield interaction (both *F* < 1; *P* > 0.20). In mGluR3^−/−^ animals, by contrast, there was a main effect of genotype (*F*_(1,30)_ = 24.78; *P* < 0.001) although no genotype–by–subfield interaction (*F* < 1; *P* > 0.20). Consistent with these findings, EAAT2 immunoreactivity was reduced in all subfields of the mGluR3^−/−^ hippocampus (dentate gyrus: *F*_(1,10)_ = 5.98; *P* = 0.035; CA3: *F*_(1,10)_ = 5.86; *P* = 0.036; CA1: *F*_(1,10)_ = 15.60; *P* = 0.003) (Table II; Fig. 1H).

### NMDA receptor subunit mRNAs

#### NR1 mRNA

In mGluR2^−/−^ mice, there was no effect of genotype on NR1 mRNA (*F* < 1; *P* > 0.20) but there was a genotype–by–subfield interaction (*F*_(2,27)_ = 3.98; *P* = 0.033). The latter reflected a small decrease in the knockouts in CA3 and an increase in CA1; however, neither change was significant in the post hoc test (CA3: *F*_(1,9)_ = 2.97; *P* = 0.119; CA1: *F*_(1,9)_ = 2.86; *P* = 0.125). NR1 mRNA was unaltered in mGluR3^−/−^ mice, with no effect of genotype or no genotype–bysubfield interaction (both *F* < 1, *P* > 0.2) (Table II).

#### NR2A mRNA

In mGluR2^−/−^ mice, there was an effect of genotype on NR2A mRNA (*F*_(1,26)_ = 4.44; *P* = 0.045), but no interaction with subfield (*F*_(2,26)_ = 2.09; *P* = 0.145). One–way ANOVA confirmed that NR2A mRNA was increased in the dentate gyrus (*F*_(1,8)_ = 10.17; *P* = 0.013), but not significantly in CA3 or CA1 (both *F* < 1; *P* > 0.20) (Table II; Figs. 1F and 3A). In mGluR3^−/−^ mice, there was again a main effect of genotype for NR2A mRNA (*F*_(1,27)_ = 11.14; *P* = 0.002), but no genotype–by–subfield interaction (*F* < 1; *P* > 0.20). Post hoc tests showed NR2A mRNA increased in the dentate gyrus (*F*_(1,9)_ = 5.73; *P* = 0.04) and CA3 (*F*_(1,9)_ = 5.35; *P* = 0.046) but not CA1 (*F*_(1,9)_ = 1.89; *P* = 0.203) of mGluR3^−/−^ mice (Table II; Fig. 3B).

#### NR2B mRNA

NR2B mRNA was unchanged in both knockout mice (all *F* < 1.7; *P* > 0.20) (Table II).

## DISCUSSION

Determining the individual properties of the two group II mGluRs, mGluR2 and mGluR3, has been problematic because of a dearth of suitable selective ligands and antibodies. The generation of mGluR2^−/−^ and mGluR3^−/−^ mice has therefore been an important step forward. Use of these mice has, for example, showed a prominent role for mGluR2 in mossy fiber long term depression (Yokoi et al., 1996) and for mGluR3 in neuroprotection (Corti et al., 2007b), and for both receptors in the anxiolytic response to a mGluR2/3 agonist (Linden et al., 2005). However, these studies have not investigated whether expression of the surviving group II mGluR, or indeed expression of other genes involved in glutamate neurotransmission, is altered in each knockout mouse. Such information is clearly relevant when interpreting their phenotype and extrapolating to the function(s) of the receptors. Here, in parallel experiments, we have studied the hippocampal expression of seven glutamatergic transcripts, and two of the encoded proteins, in mGluR2^−/−^ and mGluR3^−/−^ mice. Our results show that knockout of either gene leads to upregulation of the remaining group II mGluR and upregulation of the NR2A subunit of the NMDA receptor, as well as to downregulation of glutamate transporters. However, the identity of the affected transporter differs between the mice: loss of mGluR2 leads to a decrease of the neuronal EAAT3, whereas deletion of mGluR3 results in reduction of the glial transporters EAAT1 and EAAT2.

A notable finding was the increase of mGluR3 expression in mGluR2^−/−^ mice, and vice versa, which suggests a mutual—albeit partial—compensatory response to the loss of a group II mGluR, which may contribute to the relatively mild behavioral phenotype seen in both mice, particularly mGluR3 knockouts. Equally, the increase was only modest in percentage terms, which may explain why deletion of either gene is not without demonstrable electrophysiological and behavioral consequences (for review, see Harrison et al., in press). Complementary studies of the encoded proteins will be needed to confirm the localization and magnitude of the reciprocal upregulation.

Group II mGluRs have complex interactions with, and effects upon, NMDA receptors (Moghaddam and Adams, 1998), and it is thus of interest that, in both mGluR2^−/−^ and mGluR3^−/−^ mice, the NR2A subunit was selectively upregulated, with no alteration in either NR1 or NR2B subunit mRNAs. This finding follows a previous report that the mGluR2/3 agonist APDC enhances NMDA-evoked responses in dissociated rat prefrontal cortex neurons, predominantly via NR2A-containing receptors (Tyszkiewicz et al., 2004). Our data imply that an increased proportion of hippocampal NMDARs in mGluR2^−/−^ and mGluR3^−/−^ mice contain an NR2A rather than NR2B subunit. This difference will likely influence their properties in several ways, including calcium flux and channel opening kinetics, postsynaptic protein coupling, and perhaps long-term potentiation (Cull-Candy et al., 2001; Liu et al., 2004; Monyer et al., 1994; Sheng et al., 2000). The net functional effect of a more “NR2A–dominant” NMDA receptor population, in the presence of a lack of one or other group II mGluR, remains to be determined and will benefit from electrophysiological approaches.

A major role of group II mGluRs, especially mGluR2, is as inhibitory autoreceptors (Anwyl, 1999; Cartmell and Schoepp, 2000; Kew et al., 2002). Our results show that one consequence of deletion of either receptor is a downregulation of glutamate transporter expression. This presumably is a response to an altered level of synaptic or extra-synaptic glutamate, in turn supporting the role in vivo of both receptors in regulating synaptic glutamate release. However, three points regarding the altered EAAT expression are noteworthy. The first concerns the direction of effect. Simplistically, the loss of a group II mGluR might be predicted to increase glutamate release and thence extracellular glutamate levels, and hence that the transporters might be upregulated as part of a homeostatic response, as has been suggested for EAAT1 in cortical astrocytes (Gegelashvili et al., 1996). On the other hand, our finding is consistent with Aronica et al. (2003), who showed that mGluR3 activation increases glial glutamate transporter expression in vitro. These diverse findings emphasize that the regulation of EAAT expression and activity are complex and incompletely understood (Gegelashvili and Schousboe, 1997; Shigeri et al., 2004), and so until the effect of group II mGluR deletion on glutamate release and/or extracellular glutamate levels is known, it will remain difficult to interpret the cause and significance of the decreased glutamate transporter expression observed here.

The second point concerns the identity of the specific transporters affected in the two mouse lines. The glial glutamate transporters EAAT1 and EAAT2 were downregulated in mGluR3^−/−^ mice but unchanged in mGluR2^−/−^ mice. In contrast, the neuronal glutamate transporter EAAT3 was reduced in the mGluR2 knockouts but unchanged in their mGluR3^−/−^ counterparts. It is tempting to relate this profile to the fact that mGluR2 is exclusively neuronally expressed (Shigemoto et al., 1997), whereas mGluR3 is prominently and additionally expressed in astrocytes and other glial subtypes (Wroblewska et al., 1998). It is also of note that, in human brain, polymorphic variation in mGluR3 impacts on EAAT2 expression (Egan et al., 2004). Third, while EAAT1 mRNA and protein levels were both reduced, EAAT2 protein alone was downregulated, with EAAT2 mRNA remaining unchanged. This supports other studies showing that EAAT2 expression is regulated primarily at the translational level (Gegelashvili and Schousboe, 1997; Tian et al., 2007).

In summary, this study has shown that deletion of either mGluR2 or mGluR3 genes produces changes in the hippocampal expression of several other genes involved in glutamatergic neurotransmission. Such changes should be considered when interpreting the phenotype of the mice in behavioral, electrophysiological, and pharmacological studies. The fact that the changes are broadly similar in both mice is consistent with the grouping of mGluR2 and mGluR3 together as group II mGluRs, and for a significant overlap in their functional roles. Equally, the distinct profile of glutamate transporter alterations in mGluR2^−/−^ vs. mGluR3^−/−^ mice supports the emerging evidence for specific differences in the expression and properties of the two receptors (Corti et al., 2007b; Harrison et al., in press). Further studies need to include other brain regions, developmental stages, and additional transcripts and proteins. In particular, the expression of other glutamate receptors, most notably AMPAR subunits and additional presynaptic mGluRs, could usefully be examined. The continuing dissection of the molecular phenotype of mGluR2^−/−^ and mGluR3^−/−^ mice will contribute to the understanding of the relative contribution of each receptor in the aetiopathogenesis and pharmacotherapy of schizophrenia and other psychiatric disorders in which they are increasingly implicated.
